# Prediction of ion channel parameter differences between groups of young and aged pyramidal neurons using multi-stage compartmental model optimization

**DOI:** 10.1186/1471-2202-16-S1-P282

**Published:** 2015-12-18

**Authors:** Timothy Rumbell, Danel Draguljć, Jennifer Luebke, Patrick Hof, Christina M Weaver

**Affiliations:** 1 Fishberg Department of Neuroscience and Friedman Brain Institute, Icahn School of Medicine at Mount Sinai, New York, NY 10029, USA; 2 Department of Mathematics, Franklin and Marshall College, Lancaster, PA 17604, USA; 3 Department of Anatomy and Neurobiology, Boston University School of Medicine, Boston, MA 02118, USA

## 

Electrophysiological recording and morphological reconstruction of pyramidal neurons from layer 3 prefrontal cortex of young and aged adult rhesus monkeys reveals higher firing rates and structural differences in aged neurons relative to young [1]. Prior computational modeling of these neurons has demonstrated that morphology alone is insufficient to account for functional differences, predicting that passive and active biophysical parameters differ between age groups [2]. Here we use compartmental models featuring several independent ion channels to provide insight into the precise electrophysiological parameters that underlie excitability differences between these neurons.

We fit ion channel parameters in a neuron model using a rapid, multi-stage, semi-automated approach based on our previous optimization protocol [3], resulting in a population of models representing the target neuron. First, we use a stereotypical pyramidal cell morphology [4] with size scaled to match the target neuron surface area. Second, we use differential evolution (DE) to tune passive and H-current parameters that shape the model's response to subthreshold somatic current injection. Third, we add 7 voltage- and calcium-dependent ion channels with free maximal conductance and kinetic parameters, and set up an optimization that will fit model output to empirical responses to current injections that evoke repetitive action potential firing. We manually select error functions for the suprathreshold optimization, then use a Latin hypercube sampling (LHS) to generate a space-filling set of parameter combinations. We then simulate each point in the LHS, and use the results to calculate weights for error functions. This novel weighting method is fully automated, and scales error functions based on their expected contribution to the total error during the suprathreshold optimization. Fourth, we perform suprathreshold optimization using DE, with the free parameters and weighted error functions chosen in stage three. The result of this optimization is a population of models that fit the target neuron. DE allows for fully parallel optimization, so we generate 256 well-fitting models within 80 hours of optimization through the Neuroscience Gateway [5].

We have used this method to generate populations of models for 1 aged and 3 young neurons. Linear discriminant analysis reveals that these four populations are readily distinguished within the parameter space. Models of the three young neurons have different ion channel parameters despite similarities in electrophysiological responses. Principal component analysis (PCA) reveals that all models of the aged neuron can be separated from models of the young neurons along the first principal component. Preliminary differences between parameters fitting these four neurons reveal, for example, that in the aged neuron the L-type calcium channel activates more slowly and has greater maximal conductance, and voltage dependence of the persistent sodium channel is lower. Generation of populations of models for other empirically characterized young and aged neurons is underway with our optimization protocol. Analytical techniques such as PCA will help generate predictions about intracellular changes during normal aging.

## References

[B1] Chang YM Rosene DL Killiany RJ Mangiamele LA Luebke JI Increased action potential firing rates of layer 2/3 pyramidal cells in the prefrontal cortex are significantly related to cognitive performance in aged monkeys Cereb Cortex 2005 15 4 409 418 15749985 10.1093/cercor/bhh144

[B2] Coskren P Luebke JI Kabaso D Wearne SL Yadav A Rumbell T Hof PR Weaver CM Functional consquences of age-related morphologic changes to pyramidal neurons of the rhesus monkey prefrontal cortex J Comput Neurosci 2014 Dec 20 [Epub ahead of print] 10.1007/s10827-014-0541-5 PMC4352129 25527184

[B3] Rumbell T Draguljić D Luebke J Hof P Weaver CM Automatic fitness function selection for compartmental model optimization BMC Neuroscience 2014 15 Suppl 1 O5

[B4] Traub RD Gloveli T Whittington MA Fast rhythmic bursting can be induced in layer 2/3 cortical neurons by enhancing persistent Na+ conductance or by blocking BK channels J Neurophysiol 2003 89 909 921 12574468 10.1152/jn.00573.2002

[B5] Sivagnanam S Majumdar A Yoshimoto K Astakhov V Bandrowski A Martone ME Carnevale NT Introducing the Neuroscience Gateway IWSG, CEUR Workshop Proceedings 2013 CEUR-WS.org 10.1002/cpe.3283 PMC4624199 26523124

